# Divers risk accelerated fatigue and core temperature rise during fully-immersed exercise in warmer water temperature extremes

**DOI:** 10.1080/23328940.2019.1599182

**Published:** 2019-04-13

**Authors:** David P. Looney, Edwin T. Long, Adam W. Potter, Xiaojiang Xu, Karl E. Friedl, Reed W. Hoyt, Christopher R. Chalmers, Mark J. Buller, John P. Florian

**Affiliations:** a Biophysics and Biomedical Modeling Division, United States Army Research Institute of Environmental Medicine (USARIEM), Natick, Massachusetts, USA; b Navy Experimental Diving Unit (NEDU), Panama City, Florida, USA; c Rutgers University, School of Biomedical and Health Sciences, Newark, New Jersey, USA; d Oak Ridge Institute for Science and Education (ORISE), Oak Ridge, TN, USA

**Keywords:** Core body temperature, clothing, thermal limits, physiology, biophysics

## Abstract

Physiological responses to work in cold water have been well studied but little is known about the effects of exercise in warm water; an overlooked but critical issue for certain military, scientific, recreational, and professional diving operations. This investigation examined core temperature responses to fatiguing, fully-immersed exercise in extremely warm waters. Twenty-one male U.S. Navy divers (body mass, 87.3 ± 12.3 kg) were monitored during rest and fatiguing exercise while fully-immersed in four different water temperatures (Tw): 34.4, 35.8, 37.2, and 38.6°C (Tw_34.4_, Tw_35.8_, Tw_37.2_, and Tw_38.6_ respectively). Participants exercised on an underwater cycle ergometer until volitional fatigue or core temperature limits were reached. Core body temperature and heart rate were monitored continuously. Trial performance time decreased significantly as water temperature increased (Tw_34.4_, 174 ± 12 min; Tw_35.8_, 115 ± 13 min; Tw_37.2_, 50 ± 13 min; Tw_38.6_, 34 ± 14 min). Peak core body temperature during work was significantly lower in Tw_34.4_ water (38.31 ± 0.49°C) than in warmer temperatures (Tw_35.8_, 38.60 ± 0.55°C; Tw_37.2_, 38.82 ± 0.76°C; Tw_38.6_, 38.97 ± 0.65°C). Core body temperature rate of change increased significantly with warmer water temperature (Tw_34.4_, 0.39 ± 0.28°C·h^−1^; Tw_35.8_, 0.80 ± 0.19°C·h^−1^; Tw_37.2_, 2.02 ± 0.31°C·h^−1^; Tw_38.6_, 3.54 ± 0.41°C·h^−1^). Physically active divers risk severe hyperthermia in warmer waters. Increases in water temperature drastically increase the rate of core body temperature rise during work in warm water. New predictive models for core temperature based on workload and duration of warm water exposure are needed to ensure warm water diving safety.

## Introduction

Exercise in the heat is challenging for soldiers [1], athletes [2], and manual laborers [3,4] alike. Humans overcome heat stress in many environments primarily through evaporative cooling. However, humidity can limit the potential for evaporative heat loss through sweating. There is no evaporative underwater cooling and heat transfer from the body occurs via conduction, provided that the skin-water temperature gradient favors heat loss. The skin-water gradient narrows and eventually reverses when water temperature (Tw) increases. At the same time, increased hydrostatic pressure causes changes in circulation, renal functioning, breathing ability (on-air), and fluid shifts and fluid losses (i.e., diuresis) [5]. The addition of exercise during warm water immersion provides an opportunity to further challenge the limits of human thermoregulatory capacity.

Strenuous exercise in very warm waters is uncommon but necessary for certain military, scientific, recreational, and professional purposes. Military operations may task divers to work in the warm waters of the Persian Gulf; where sea surface temperatures typically reach 35°C but have increased by nearly 0.6°C since 1990 [6]. Microbiological research on thermophilic bacteria has involved scuba diving expeditions to shallow hydrothermal fissures venting water at temperatures between 30–95°C [7]. Tourists scuba dive in natural hot springs such as the Homestead Crater in Utah where water temperature is approximately 35°C [8]. The Dead Sea is frequently visited by both researchers [9] and tourists [10] and has sea surface temperatures that have historically ranged between 33–36°C during the summer months [11]. Furthermore, daytime sea surface temperatures in the Dead Sea were shown to have increased by 0.6°C·decade^−1^ over the period of 2000–2016 [12]. Thermoregulatory responses to warm water exercise must be better understood for the safety of current divers and future generations that face rising global water temperatures.

The effects of cold water immersion have been extensively researched [13–16] but fewer studies have examined the physiological and thermoregulatory responses to warm immersion exercise. Previous studies have examined exercise with either mid-chest [17] or heads out [18] immersion, following pre-exercise immersion [19], or while wearing a water-perfusion jacket [20]. The level of water immersion (e.g. knee, hip, chest) results in varying responses in physiological measures such as heart rate and oxygen uptake [21]. However, the physiological responses to full warm water immersion have not been fully elucidated. This study examined core temperature (Tc) and cardiovascular responses to fatiguing, fully-immersed exercise in extremely warm waters in order to better understand human thermoregulatory limits. We hypothesized that the rate of change in Tc (ROC.Tc) would increase significantly with warmer Tw.

## Methods

### Participants

Twenty-one male Navy-trained divers (body mass, 87.3 ± 12.3 kg) from the Navy Experimental Diving Unit (NEDU) and the Navy Diving and Salvage Training Center volunteered for this investigation. All participants provided voluntary informed consent before performing any experimental procedures. The study was approved by the review committee for the protection of human subjects at NEDU and the Institutional Review Board at the U.S. Army Research Institute of Environmental Medicine (USARIEM; Natick, MA).

### Procedures

Prior to data collection, all participants completed a five week periodized cycle ergometer conditioning program in the NEDU Environmental Chamber (room temperature, 34.4°C; relative humidity, 50%) and an initial 4 h familiarization exercise dive with a water temperature (Tw) of 25.6°C. The familiarization dive followed the same protocol as the test exercise dives and was designed to expose participants to testing procedures, reduce learning effects, and ensure divers could tolerate the physical workload of the exercise test in the absence of thermal strain from the warmer water conditions. Subsequently, participants completed both a resting dive (≤ 8 h) and an exercise dive (≤ 4 h) at four different Tw in ascending order: 34.4, 35.8, 37.2, and 38.6°C (Tw_34.4_, Tw_35.8_, Tw_37.2_, and Tw_38.6_ respectively). Treatment order was not randomized due to diver safety concerns based on studies completed at the Naval Medical Research Center (NRMC) [22]. Familiarization, resting, and exercise dives were separated by at least 3 days of recovery.

Participants consumed one Meal, Ready-to-Eat (MRE) and at least 1 L of water the evening before scheduled dives and refrained from consuming either alcohol (> 48 h) and caffeine (> 24 h). On the morning of each dive, participants reported to the Physiology Lab two hours prior to their scheduled start times and were provided another MRE and 0.3 L of water. Diver dress consisted of a cotton T-shirt, swim trunks, and diver booties. Participants were weighed immediately before and after they entered the water for each dive to determine body mass changes (Post – Pre) for rehydration purposes. Body mass changes are reported for descriptive purposes but should not be interpreted as solely indicative of sweat loss since divers were permitted to urinate during the test dives and urine volume measurements were not available for analysis.

Core temperature (Tc) was recorded every 30-sec using an YSI 700 series thermistor probe (YSI, Inc.; Yellow Springs, OH). This probe was inserted 15 cm past the anal verge and retained there by a 6.4 mm diameter button. Water temperature was monitored using another YSI 700 series thermistor probe positioned at the participants’ depth and remained within ± 0.28°C of the designated Tw. Core temperature and Tw were recorded every 30 seconds with LabVIEW (National Instruments; Austin, TX) software on an NEDU data acquisition system (DAS) computer on the test pool medical deck. Heart rate was continuously monitored with Quinton Q-Tel Rehab ECG telemetry (Quinton Cardiology Systems, Inc.; Bothell, WA) and manually logged every five minutes. Two trolling motors (MotorGuide, WI; Minn Kota, MN) stirred the water column in the test pool to ensure a more homogeneous Tw. Oxygen pressure gauges and thermistors were calibrated at the beginning of each day.

Participants donned the underwater breathing apparatus (OXY-LUNG UBA; Aqua Lung America, Inc.; Vista, CA) and entered the test pool after all instrumentation checks were completed. Each participant then boarded a Modified Collins Pedal-Mate cycle ergometer (Collins Medical; Braintree, MA) set at zero inclination on a platform approximately three feet deep. Participants remained approximately 1–2 feet below the water surface in a slightly upward prone position with shoulders pressed against restraints, each hand gripping a handle, and feet strapped to the cycle ergometer for the entire dive. An Extrel Mass Spectrometer Model GS (ABB Extrel; Pittsburgh, PA) monitored inspired gas concentrations during all dives to ensure participant safety. Participants were asked to pedal at 60 rpm at a level of resistance that produced a work rate equal to a standard combat swim (50 watts) [23]. Trials were terminated when (a) the participant was unable to sustain the work rate; (b) Tc exceeded 40°C for 5 min; (c) Tc exceeded 40.5°C at any time; (d) if the participant requested termination for any reason; or (e) after 4 hours.

### Data analysis

All data were analyzed using analyzed using R (Version 3.3.1; R Foundation for Statistical Computing; Vienna, Austria) [24]. Data are displayed as mean ± SD unless specifically noted otherwise. Rate of change in core temperature (ROC.Tc) was calculated as the average rate of change in core temperature over time. Pairwise least-squares mean comparisons were conducted using mixed effects models with random intercepts if a significant main effect was detected by analysis of variance. The level of statistical significance was set at p < 0.05.

## Results

**Figure 1.**
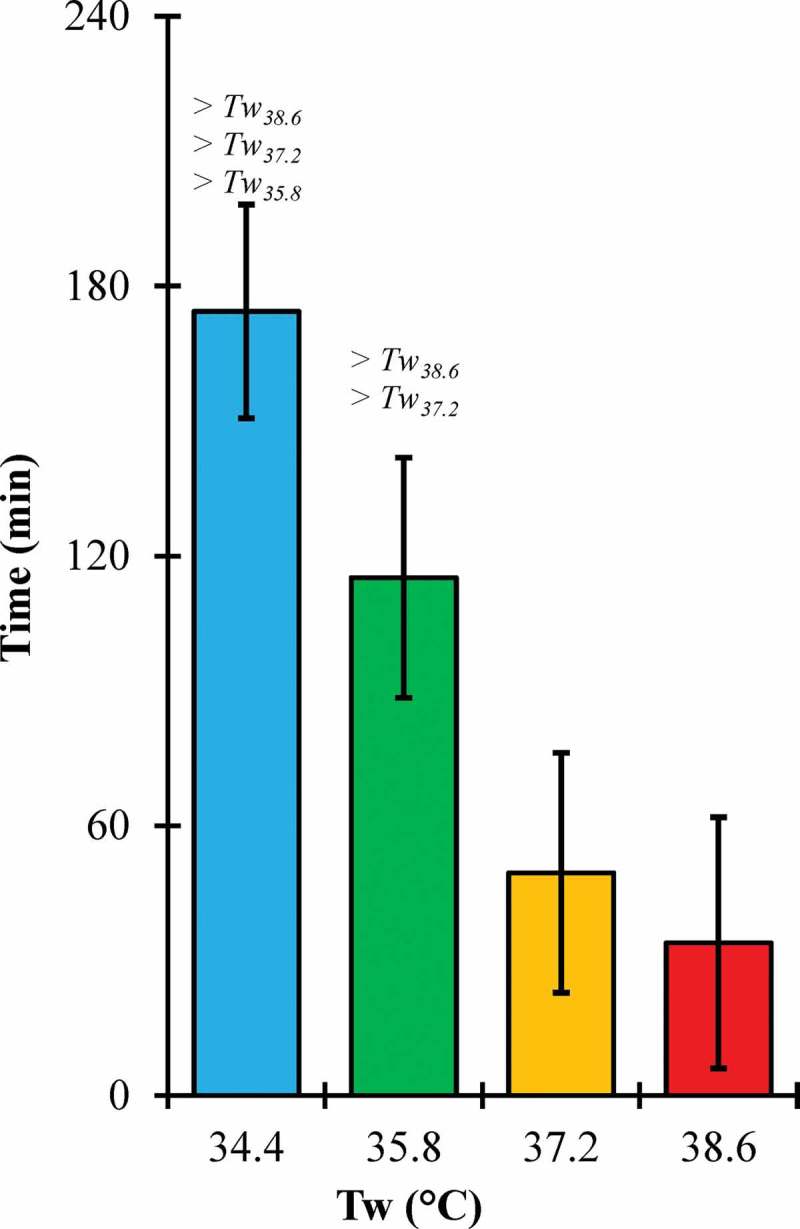
Endurance time across water temperature (Tw) conditions. Error bars, 95% confidence intervals; > #, significantly greater than #°C water condition.

There was a significant main effect of Tw on trial endurance time (p < 0.001) (Figure 1). Trial time was significantly longer for Tw_34.4_ versus the Tw_35.8_, Tw_37.2_, and Tw_38.6_ conditions (p < 0.001 for each). Similarly, trial time was significantly higher for Tw_35.8_ versus Tw_37.2_ (p = 0.001) and Tw_38.6_ (p < 0.001). However, there was no significant difference between Tw_37.2_ and Tw_38.6_ (p = 0.409). Four participants reached the 4-hour time limit for Tw_34.4_ while only one finished for Tw_35.8_. Two trials were halted due to Tc limits for the Tw_37.2_ and Tw_38.6_ conditions with none of the participants reaching the 4-hour time limit in either condition. There was no significant main effect of Tw on body mass change (p = 0.061). Body mass changes were negative; indicating body mass was lost during test dives (Tw_34.4_, −1.8 ± 0.9%; Tw_35.8_, −2.5 ± 1.3%; Tw_37.2_, −2.0 ± 1.2%; Tw_38.6_, −1.4 ± 0.9%).

**Figure 2.**
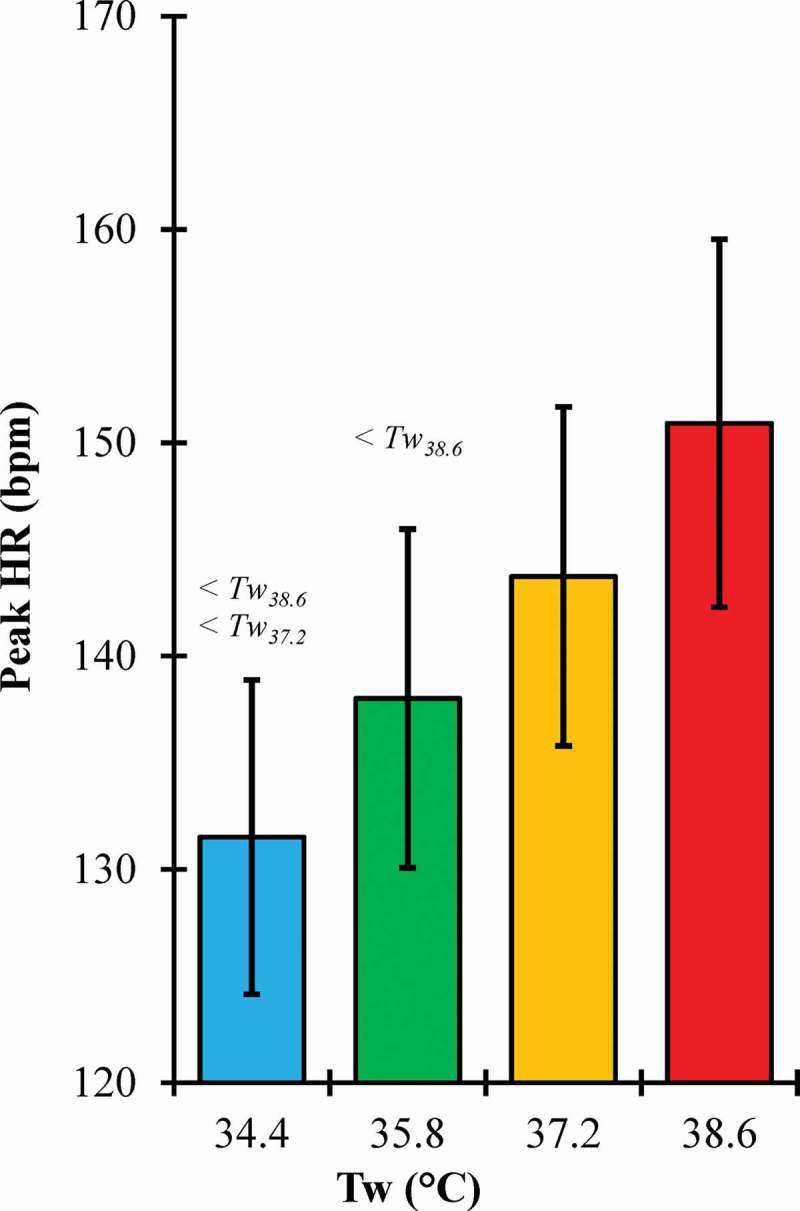
Peak heart rate (HR) across water temperature (Tw) conditions. Error bars, 95% confidence intervals; < #, significantly lower than #°C water condition.

There was a significant main effect of Tw on peak HR (p = 0.002) (Figure 2). Peak HR for Tw_34.4_ was not significantly different from Tw_35.8_ (p = 0.107) but was significantly lower than Tw_37.2_ (p = 0.007) and Tw_38.6_ (p < 0.001). Similarly, peak HR for Tw_35.8_ was not significantly different than Tw_37.2_ (p = 0.231) but was significantly lower than Tw_38.6_ (p = 0.016). However, peak HR for Tw_37.2_ was not significantly different than Tw_38.6_ (p = 0.165).

**Figure 3.**
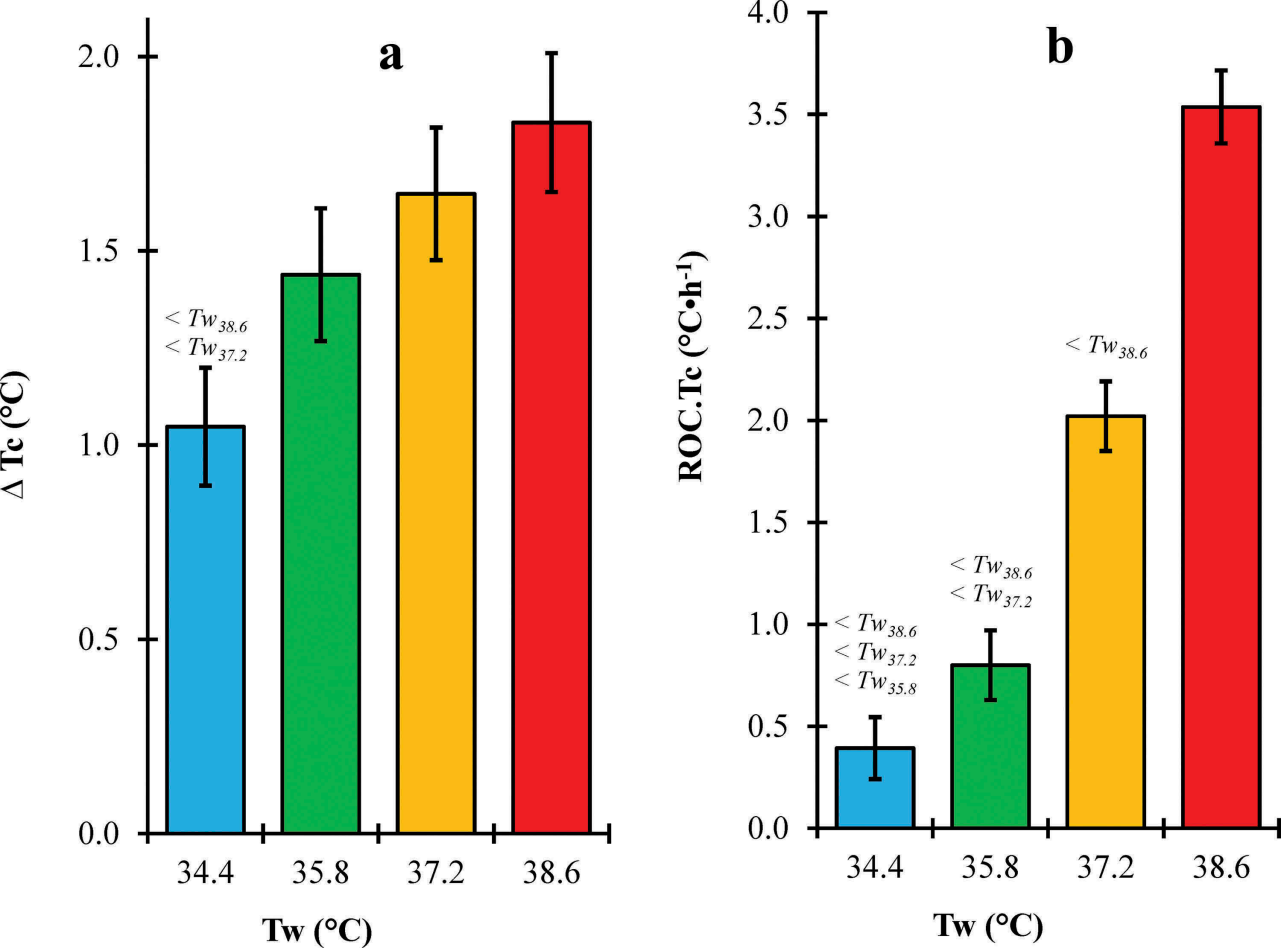
∆Tc (a) and ROC.Tc (b) across water temperature (Tw) conditions. Tc, core temperature (°C); ROC.Tc, rate of change in core temperature (°C·h^−1^); Error bars, 95% confidence intervals; < #, significantly lower than #°C water condition.

There was a significant main effect of Tw on peak Tc (p = 0.038) but not initial Tc (p = 0.329) (Figure 3). Peak Tc for Tw_34.4_ was not significantly different from Tw_35.8_ (p = 0.203) but was significantly lower than Tw_37.2_ (p = 0.032) and Tw_38.6_ (p = 0.008). In contrast, peak Tc for Tw_35.8_ was not significantly different than Tw_37.2_ (p = 0.361) or Tw_38.6_ (p = 0.145). Peak Tc for Tw_37.2_ was not significantly different than Tw_38.6_ (p = 0.547).

**Figure 4.**
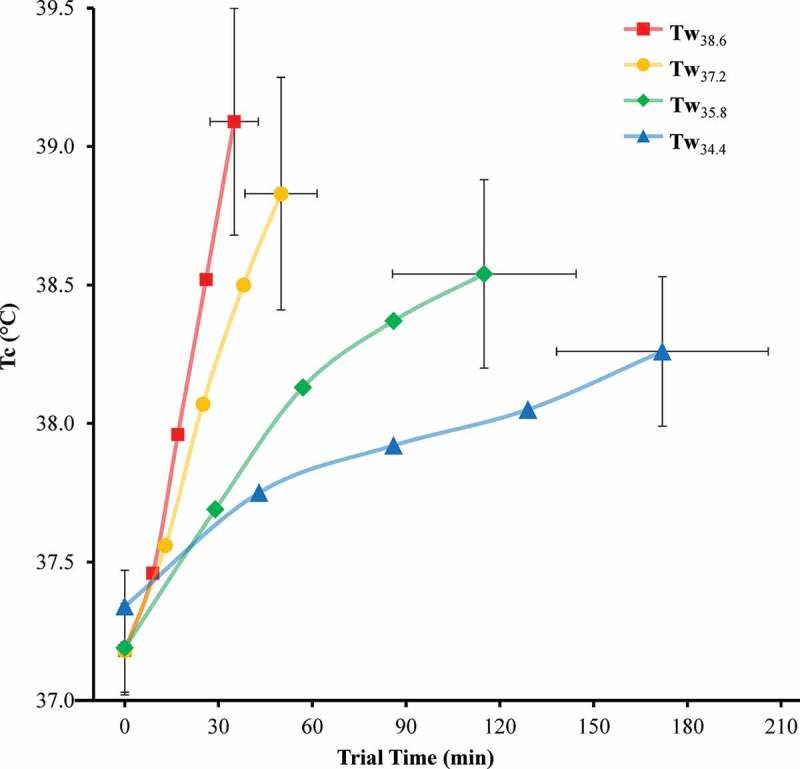
Trial endurance time and core temperature (Tc) at 0, 25, 50, 75, and 100% trial time. Error bars, 95% confidence intervals.

**Figure 5.**
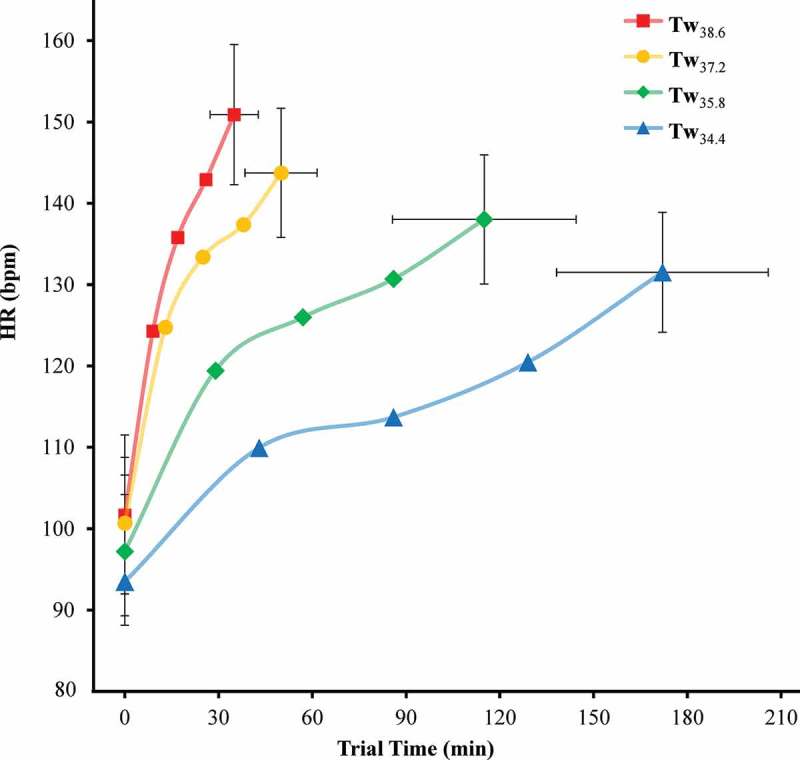
Trial endurance time and heart rate (HR) at 0, 25, 50, 75, and 100% trial time. Error bars, 95% confidence intervals.

There was a significant main effect of Tw on ∆Tc (p = 0.003) and ROC.Tc (p < 0.05 for each) (Figure 4). ∆Tc for Tw_34.4_ was significantly lower than Tw_37.2_ (p = 0.004) and Tw_38.6_ (p < 0.001) but not Tw_35.8_ (p = 0.064). ∆Tc for Tw_35.8_ was not significantly different than Tw_37.2_ (p = 0.342) or Tw_38.6_ (p = 0.097). ∆Tc for Tw_37.2_ was not significantly different than Tw_38.6_ (p = 0.435). ROC.Tc for Tw_34.4_ was significantly lower than Tw_35.8_, Tw_37.2_, and Tw_38.6_ (p < 0.001 for each). Similarly, ROC.Tc for Tw_35.8_ was significantly lower than Tw_37.2_ and Tw_38.6_ (p < 0.001 for each). Furthermore, ROC.Tc for Tw_37.2_ was also significantly lower than Tw_38.6_ (p < 0.001). Figures 4 and 5 display Tc and heart rate respectively over trial endurance time for each Tw condition.

## Discussion

Warm water immersion results in increasingly rapid rises in Tc and onset of fatigue during exhaustive exercise. Rate of change in Tc increased significantly with warmer Tw indicating an unsustainable level of thermal-work strain. Additionally, there was a drastic reduction in trial endurance time in the warmer Tw conditions. Although all participants were required to complete a 4 h exercise dive in 25.6°C water before testing, the same work rate was only sustained for 34 ± 14 min in the Tw_38.6_ condition. These findings highlight the dangers of warm water diving expeditions and enhance understanding of human thermoregulatory limits.

Prior investigations have noted reduced aerobic endurance capacity with warm water immersion [25,26]. Nybo et al. [27] reviewed a combination of different cardiovascular, psychological, neurobiological, respiratory, and muscular factors that explain the accelerated fatigue during exertional heat stress [27]. Temperature-related reductions in time trial performance have been attributed to impaired nerve conduction and force development [28]. Exertional hyperthermia decreases skeletal muscle activation [29] while increasing perceived exertion [30]. Watson et al. [19] noted significantly greater perceived exertion in addition to elevated heart rate, blood glucose, and lactate concentrations during exercise in 39°C water versus 35°C. In the current study, participants were likely unable to regulate Tc in the warmer Tw conditions due to the loss of sweat evaporation capabilities and heat gained from the water.

The results in Figure 4 show that heat stress during warm water immersion dramatically accelerates ROC.Tc during exercise. Few studies have observed such extreme rates of Tc rise as the present study (3.54°C·h^−1^ for Tw_38.6_) during strenuous exercise in warm water environments where the body gains heat from the water. Gonzalez-Alonso et al. [20] noted a rate of change in esophageal temperature of 6.31°C·h^−1^ in trained cyclists during an endurance trial (258 ± 20 W; 87 ± 2 rpm; 66 ± 3% VO_2_ max) while wearing a 42°C water-perfusion jacket in a 40°C environmental chamber. Rhind et al. [17] observed an average ROC.Tc of 3.15°C·h^−1^ after 40 min of cycle ergometer exercise (65% VO_2_ peak) while immersed to mid-chest in 39°C water. In contrast, Macaluso et al. [31] recorded an average ROC.Tc of only 0.87°C·h^−1^ in competitive masters swimmers following a 5-km simulated race in 32°C water.

One limitation of this investigation is that treatment order was not randomized. Trials were scheduled in ascending order of Tw and were scheduled at least 3 days after a resting trial at the designated Tw to best ensure diver safety and minimize heatstroke risk. Acclimatization status is a prominent moderator of the effect of environmental heat stress on endurance exercise performance [32]. Consequently, the treatment order may have blunted the thermoregulatory responses observed in the higher Tw conditions. Another limitation for this investigation is the lack of skin temperature data which is essential for tracking the time course of the inversion of the skin-water temperature gradient. High skin temperatures are associated with earlier exhaustion during submaximal exercise in the heat [33–35]. This performance decrement has been attributed to reductions in cardiac output and peak oxygen uptake resulting from increased peripheral blood flow [33].

This study examined Tw conditions that are greater or equal to the most extreme warm water environments where diving occurs outside of the laboratory. The divers faced a level of exertional heat stress that is unrealistic for the majority of everyday scenarios. The highest Tw conditions (Tw_37.2_ and Tw_38.6_) exceed nearly all current warm water diving environments outside of the laboratory. However, climate change poses major heat illness risks [36] and rising global Tw trends [6,12,37] suggest divers may be exposed to greater thermal stress over time. These health risks may be exacerbated in the aging workforce due to the reduced ability of older adults to compensate for heat gain [38]. This study provides insight on the excessive thermal-work strain experienced during fatiguing warm water exercise that is of relevance to military, recreational, and professional divers who will endure similar conditions in the near or distant future.

## Conclusion

Divers risk severe predictable hyperthermia in warmer waters. Physical performance degrades sharply at warmer temperature extremes. Incremental increases in water temperature drastically increase the rate of core temperature rise. Precise core temperature monitoring strategies and/or predictive models are needed to ensure warm water diving safety.
